# Numerical simulation on transient electromagnetic response of separation layer water in coal seam roof

**DOI:** 10.1038/s41598-024-66529-5

**Published:** 2024-07-09

**Authors:** Haiyan Yang, Junjun Jiao, Qiang Wang, Zhixin Liu, Benyu Su, Yunlei Xu, Wenyu Li, Huageng Ran

**Affiliations:** 1 School of Resources and Geosciences, China University of Mining and Technology, Xuzhou, 221116 Jiangsu China; 2 College of Earth Science and Surveying Engineering, China University of Mining and Technology-Beijing, Beijing, 100083 China; 3 WA School of Mines: Minerals, Energy and Chemical Engineering, Curtin University, Perth, WA Australia; 4 Technology Center of Jinneng Holding Coal Industry Group Co., Ltd., Datong, 037003 Shanxi China

**Keywords:** Mine transient electromagnetic method, Separation layer water, Time-domain finite-difference method, Numerical simulation, Geology, Geophysics

## Abstract

Mining stress induces deformation and fracture of the overlaying rock, which will result in water filling the separation layer if the aquifer finds access to abscission space along the fracture channels. Accurate detection is crucial to prevent water hazards induced by water-bearing fractures. The 3-D time-domain finite-difference method with Yee’s grid was adopted to calculate full-space transient electromagnetic response; meanwhile, a typical geologic and geophysical model with a water-bearing block in an separation layer was built according to regional tectonics and stratigraphic developments. By using numerical simulation, the induced voltage and apparent resistivity for both vertical and horizontal components were acquired, and then an approximate inversion was carried out based on the “smoke ring” theory. The results indicate that the diffusion velocity of induced voltage is significantly affected by the water-bearing body in the fracture, and the horizontal velocity of induced voltage is lower than the vertical one. The induced voltage curves indicate that the horizontal response to an anomaly body is stronger than the vertical one, leading to a high apparent resistivity resolution of conductivity contrast and separation layer boundary in the horizontal direction. The results of 3-D simulation making use of a measured data model also demonstrate that the horizontal component of apparent resistivity can reflect the electrical structure in a better way; however, its ability to recognize the concealed and fine conductor is rather weak. Accordingly, the observation method or numerical interpolation method needs to be further improved for data processing and interpretation.

## Introduction

Transient electromagnetic method (TEM) is a remote sensing technology based on electromagnetic induction, and it is also one of the most effective methods for near-surface geophysical exploration^1^. With the rapid development of transient electromagnetic remote sensing instruments and interpretation methods, the application scenario of TEM has shifted from the ground to the underground space of coal mines.

During deep coal seam mining, the structure, strength, and stress environment of the weak mudstone in the roof and floor undergo alterations due to mining stress. This results in key strata deformation and the formation of numerous longitudinal fissures post-breakage. These fissures connect with the aquifer of the rock stratum, leading to water filling the separation space. They exhibit characteristics such as large instantaneous water volume, strong periodicity, and no obvious water inrush symptoms^2,3^. Considering the issue of coal mine separation layer water disasters, studying the geophysical field characteristics of separation layer water richness is crucial. This aspect plays a vital role in uncovering the formation mechanism and preventing coal mine separation layer water disasters. Accurately locating the separation layer water in the roof and floor of the coal seam is paramount in preventing separation layer water damage. Typically, separation layer water is found several hundred meters underground. Preventing and controlling such water damage primarily relies on drilling and geophysical exploration technology. Initially, geophysical exploration technology is utilized to delineate the water-rich area of separation layer, followed by drilling technology for drainage or plugging^4^. Research indicates that both the DC resistivity method and the transient electromagnetic method significantly aid in detecting water-rich strata. The transient electromagnetic method stands out for its simplicity, high construction efficiency, and superior resolution in detecting water-rich areas compared to the DC resistivity method. Currently, it is the preferred method for detecting water disasters in mine roof and floor areas^5–7^.

In terms of the geophysical detection method of separation layer water, Li et al.^8^ used borehole television (TV) imaging detection technology in a coal mine to obtain the distribution and hydrogeological features of the “water accumulation separation zone” and “vacuum separation zone” in the overlying rock of the working face. Li et al.^9^ used transient electromagnetic methods and borehole TV imaging technology to investigate the separation layer water and considered that the soft and hard combination of sandstone and mudstone was easily affected by mining to form the separated layer space. Zhang et al.^10^ proposed monitoring the failure height of the overlying rock in the working face by using the borehole-tunnel resistivity method and obtaining the height of the “two zones”. In addition, Qiao et al.^2^ used the key layer discrimination formula to calculate the approximate development horizon of the separation layer development and then employed the drilling method to determine the exact horizon of the separation layer development. Su et al.^11^ used the down-hole transient electromagnetic method to dynamically monitor the electromagnetic response characteristics of separation layer water before, during and after coal seam mining, and summarized the development law of separation layer water. In the above research, the drilling method has a large amount of construction work and high detection cost; the borehole TV imaging technology has the limitation of “one-hole view” and it is difficult to obtain the boundary position of the separation layer. Although the direct current resistivity method can be used to monitor the development horizon of the separated layer water, it is seriously affected by the volume effect in the coal mine. The application of mine transient electromagnetic method in the detection of separation layer water has the advantages of high accuracy and construction efficiency. Therefore, the three-dimensional numerical simulation technology of mine transient electromagnetic method is considered to study the response characteristics of separation layer water in coal mine.

The 3-D forward numerical calculation methods of the transient electromagnetic method mainly include the finite difference method, integral equation method, and finite element method^12^. With the application of TEM from the ground to the full underground space, domestic scholars have gradually applied the above numerical methods to mine TEM numerical simulation research, with the finite difference method as the dominant one. Yue et al.^13^ carried out a full-space three-dimensional numerical simulation study on the low-resistance abnormal body of the roadway floor by using the finite difference time domain method. Yang^14^ simulated the transient electromagnetic field of the small loop source in the whole space based on the finite difference time domain method and studied the influence of the roadway on the transient electromagnetic field of the whole space. Based on Yee’s grid division time-domain finite difference method, Chang et al.^15^ simulated and studied the full-space transient electromagnetic field response law of water-bearing subsidence columns. Zhang et al.^16^ studied the three-dimensional full-space response characteristics of vector finite element mine transient electromagnetics and discussed the shut-off effect, the influence of roadway, and the response law in the case of water accumulation and goaf. Based on the finite volume method, Zhou et al.^17^ explored the interference effect of metal body in tunnel on TEM data and proposed a correction method under the interference of metal body. Chen et al.^18^ used the integral equation method to simulate the multi-component transient electromagnetic response characteristics of the coal seam floor aquifer and discussed the response law of the horizontal and vertical components observed in the borehole. Currently, mine TEM 3-D forward modeling is still in the theoretical research stage. In comparison, the detection methods based on the development of mine TEM 1-D forward modeling are relatively mature and complete. For the water-rich detection of mine rock mass, the mine transient electromagnetic method is mostly used to detect the water-richness on the roof, floor, and head-on face of the working face, which greatly ensures the safe mining during the excavation process of the working face^19–22^. In addition, great progress has also been made in terms of device form^23^, off-time correction^24^, and time-depth conversion method^25^.

At present, most of the forward and inversion studies on mine transient electromagnetic methods focus on mine water-conducting structures (water-conducting faults, water-filled subsidence columns, water-filled goafs, aquifers, and fissure water). However, the studies on the transient electromagnetic field response law of separation layer water in coal seam roofs are comparatively insufficient. Aiming at the above problems, this study established a geological-geophysical model of the roadway roof containing separation layer water and used the three-dimensional FDTD method to explore the multi-component diffusion law of the transient electromagnetic field of separation layer water in the roadway roof. On this basis, a full-space three-dimensional forward model containing separation layer water was developed, and the multi-component TEM response characteristics of the low-resistance anomalous body with separation layer water in the roadway roof were verified and analyzed.

## Theoretical basis

### Maxwell’s equations and 3-D difference equations

The underground transient electromagnetic field excited by the emission field source follows Maxwell’s equations of electromagnetic field:

1 $$\nabla \times {\varvec{E}} = - \frac{{\partial {\varvec{B}}}}{\partial t}$$

2 $$\nabla \times {\varvec{H}} = \frac{{\partial {\varvec{D}}}}{\partial t} + {\varvec{D}} + {\varvec{J}}$$

3 $$\nabla \cdot {\varvec{B}} = 0$$

4 $$\nabla \cdot {\varvec{D}} = \rho$$

and thus satisfies the following relation:

5 $${\varvec{J}} = \sigma \user2{E,}\;\;{\varvec{B}} = \mu {\varvec{H}},\;\;{\varvec{D}} = \varepsilon {\varvec{E}}$$

In Formula (5), ***E*** and ***H*** denote the electric and magnetic field strengths (*V/m* and *A/m*), respectively; ***B*** and ***D*** denote the magnetic induction strength (*T*) and electric displacement vector (*C/m*^*2*^), respectively; ***J*** refers to conduction current density (*A/m*^*2*^); ρ is charge density (*C/m*^*3*^); μ, ε, σ are permeability (*H/m*), permittivity (*F/m*) and conductivity (*S/m*).

**Figure 1:**
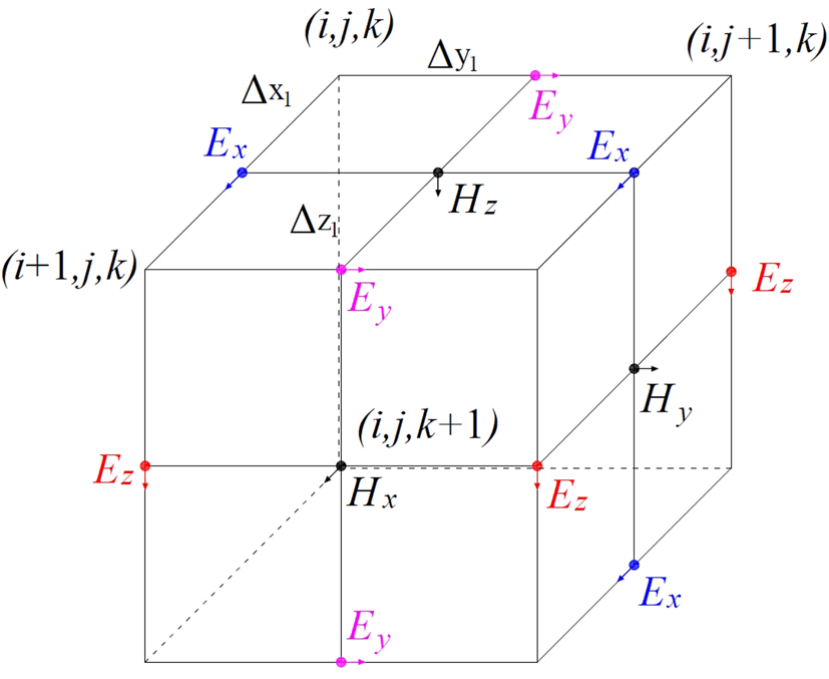
Yee unit cell meshing.

The model space is meshed with Yee's grid (Fig. 1), and the above Maxwell equation (ignoring the displacement current term) is solved by the finite difference method^13^, and the difference equation is as follows:

6 $$\begin{aligned} E^{n + 1} \left( {i,j,k} \right) & & = \frac{1}{{r\left( {i,j,k} \right)}}\left[ {a^{\prime}\left( {i,j,k} \right)E^{n - 1} \left( {i,j,k} \right)} \right. \\ & \;\; + a_{1}^{\prime } \left( {i,j,k} \right)E^{n} \left( {i + 1,j,k} \right) \\ & \;\; + a_{2}^{\prime } \left( {i,j,k} \right)E^{n} \left( {i - 1,j,k} \right) \\ & \;\; + a_{3}^{\prime } \left( {i,j,k} \right)E^{n} \left( {i,j + 1,k} \right) \\ & \;\; + a_{4}^{\prime } \left( {i,j,k} \right)E^{n} \left( {i,j - 1,k} \right) \\ & \;\; + a_{5}^{{\prime {^{\prime}}}} \left( {i,j,k} \right)E^{n} \left( {i,j,k + 1} \right) \\ & \;\; + a_{6}^{\prime } \left( {i,j,k} \right)E^{n} \left. {\left( {i,j,k - 1} \right)} \right] \\ \end{aligned}$$

In Formula (6):

7 $$\left\{ \begin{gathered} r\left( {i,j,k} \right) = b\left( {i,j,k} \right) - a\left( {i,j,k} \right){\Delta }t \hfill \\ a^{\prime}\left( {i,j,k} \right) = b\left( {i,j,k} \right) + a\left( {i,j,k} \right){\Delta }t \hfill \\ a^{\prime}_{n} \left( {i,j,k} \right) = 2a_{n} \left( {i,j,k} \right){\Delta }t,n = 1,2, \ldots n \hfill \\ \end{gathered} \right.$$

In Formula (7):

8 $$\left\{ \begin{gathered} a_{1} \left( {i,j,k} \right) = \frac{{\left( {{\Delta }y_{j} + {\Delta }y_{j + 1} } \right)\left( {{\Delta }z_{k} + {\Delta }z_{k + 1} } \right)}}{{4{\Delta }x_{i} }} \hfill \\ a_{2} \left( {i,j,k} \right) = \frac{{\left( {{\Delta }y_{j} + {\Delta }y_{j + 1} } \right)\left( {{\Delta }z_{k} + {\Delta }z_{k + 1} } \right)}}{{4{\Delta }x_{i + 1} }} \hfill \\ a_{3} \left( {i,j,k} \right) = \frac{{\left( {{\Delta }x_{i} + {\Delta }x_{i + 1} } \right)\left( {{\Delta }z_{k} + {\Delta }z_{k + 1} } \right)}}{{4{\Delta }y_{j} }} \hfill \\ a_{4} \left( {i,j,k} \right) = \frac{{\left( {{\Delta }x_{i} + {\Delta }x_{i + 1} } \right)\left( {{\Delta }z_{k} + {\Delta }z_{k + 1} } \right)}}{{4{\Delta }y_{j + 1} }} \hfill \\ a_{5} \left( {i,j,k} \right) = \frac{{\left( {{\Delta }x_{i} + {\Delta }x_{i + 1} } \right)\left( {{\Delta }y_{j} + {\Delta }y_{j + 1} } \right)}}{{4{\Delta }z_{k} }} \hfill \\ a_{6} \left( {i,j,k} \right) = \frac{{\left( {{\Delta }x_{i} + {\Delta }x_{i + 1} } \right)\left( {{\Delta }y_{j} + {\Delta }y_{j + 1} } \right)}}{{4{\Delta }z_{k + 1} }} \hfill \\ a\left( {i,j,k} \right) = - \left[ \begin{gathered} a_{1} \left( {i,j,k} \right) + a_{2} \left( {i,j,k} \right) + a_{3} \left( {i,j,k} \right) + \hfill \\ a_{4} \left( {i,j,k} \right) + a_{5} \left( {i,j,k} \right) + a_{6} \left( {i,j,k} \right) \hfill \\ \end{gathered} \right] \hfill \\ \end{gathered} \right.$$

9 $$\begin{aligned} b\left( {i,j,k} \right) & = \frac{\mu }{8}\left[ {\sigma \left( {i,j,k} \right)} \right.{\Delta }x_{i} {\Delta }y_{j} {\Delta }z_{k} \\ & \;\; + \sigma \left( {i + 1,j,k} \right){\Delta }x_{i + 1} {\Delta }y_{j} {\Delta }z_{k} \\ & \;\; + \sigma \left( {i,j + 1,k} \right){\Delta }x_{i} {\Delta }y_{j} {\Delta }z_{k} \\ & \;\; + \sigma \left( {i,j,k + 1} \right){\Delta }x_{i} {\Delta }y_{j} {\Delta }z_{k + 1} \\ & \;\; + \sigma \left( {i + 1,j,k + 1} \right){\Delta }x_{i + 1} {\Delta }y_{j} {\Delta }z_{k + 1} \\ & \;\; + \sigma \left( {i + 1,j + 1,k} \right){\Delta }x_{i + 1} {\Delta }y_{j + 1} {\Delta }z_{k} \\ & \;\; + \sigma \left( {i,j + 1,k + 1} \right){\Delta }x_{i} {\Delta }y_{j + 1} {\Delta }z_{k + 1} \\ & \;\; + \sigma \left( {i + 1,j + 1,k + 1} \right){\Delta }x_{i + 1} {\Delta }y_{j + 1} {\Delta }z_{k + 1} \\ \end{aligned}$$

### Initial field source and boundary conditions

When *t* = *t*_*0*_, *t*_*1*_ > 0, the uniform full-space magnetic dipole source is transformed into the initial condition and added; *t*_*0*_ and *t*_*1*_ should be made as small as possible. As for the parameter selection that affects the computational efficiency and accuracy, the relationship between time and space steps in the literature is still used^13^. The magnetic dipole source is placed along the direction of *x*, and the electric field generated by the sudden interruption of the electric current in the galvanic source under full space conditions is as follows^26^:

10 $$\begin{aligned} E & = \frac{m}{{4\pi r^{3} }}\left\{ {\left[ {\left( {\frac{4}{\sqrt \pi }\theta^{3} r^{3} + \frac{6}{\sqrt \pi }\theta r} \right)} \right.} \right.{\text{e}}^{{ - \theta^{2} r^{2} }} \\ & \;\; + 3Er\left. {f\left( {\theta r} \right)} \right] \times \left( {\frac{{x^{2} }}{{r^{2} }}u_{x} + \frac{xy}{{r^{2} }}u_{y} + \frac{xz}{{r^{2} }}u_{z} } \right) \\ & \;\; - \left[ {\left( {\frac{4}{\sqrt \pi }\theta^{3} r^{3} + \frac{2}{\sqrt \pi }\theta r} \right){\text{e}}^{{ - \theta^{2} r^{2} }} + Erf\left( {\theta r} \right)} \right]\left. {u_{x} } \right\} \\ \end{aligned}$$

11 $$\theta = \left( {\frac{{\mu_{\sigma } }}{4t}} \right)^{\frac{1}{2}} ,\;\;r = \left( {x^{2} + y^{2} + z^{2} } \right)^{\frac{1}{2}}$$

In Formula (10), $m$ denotes the magnetic moment; $r$ is the transmitter–receiver distance; and ${\text{erf}}\left( {\theta r} \right)$ represents the error function.

As for the determination of electromagnetic field boundary conditions, Liao’s absorption boundary conditions have higher computational accuracy^27^. Once improved by high-order correction, the conditions would display a better processing effect of boundary corners, especially for the processing of roadway boundaries.

### Apparent resistivity calculation and “smoke ring” inversion

The apparent resistivity conversion formula of the vertical component of the induced electromotive force is expressed as follows^14^:

12 $$\rho_{{\text{s}}} \left( z \right) = \frac{\mu }{4\pi t}\left( {\frac{{\mu S_{T} S_{R} }}{t}} \right)^{2/3} \left( {\frac{{V_{z} \left( t \right)}}{I}} \right)^{ - 2/3}$$

In Formula (12), *S*_*T*_ and *S*_*R*_ denote the areas of the transmitting and receiving coils respectively; *I* is the transmitting current; $V_{z} \left( t \right)$ refers to the induced electromotive force in the vertical direction; and *t* represent the sampling time.

To calculate the apparent resistivity of the horizontal component of the induced electromotive force, the definition method in the literature is adopted^28^, namely:

13 $$\rho_{{\text{s}}} \left( {\text{x}} \right) = \frac{{\mu_{0} }}{\pi t}\left[ {\frac{{C_{{\text{r}}} \pi I\mu_{0} }}{{4tV_{x} \left( t \right)}}} \right]^{\frac{2}{3}}$$

14 $$C_{{\text{r}}} = \frac{{C_{{{\text{r}}2}} }}{8} - \frac{{C_{{{\text{r}}1}} }}{40} - \frac{{C_{{{\text{r}}3}} }}{2}$$

In Formulas (13, 14), $V_{{\text{x}}} \left( t \right)$ denotes the induced electromotive force in the horizontal direction; and $C_{{{\text{r}}1}}$ $C_{{{\text{r}}2}}$ $C_{{{\text{r}}3}}$ are the horizontal component fitting polynomial coefficients, their values can be referred to [28].

The “smoke ring” inversion is an approximate inversion method that does not require an initial model. For horizontal layered media, the “smoke ring” inversion mainly consists of three steps:

(1) To find the eddy current diffusion velocity

15 $$V = \frac{4}{{\sqrt {\pi \mu_{0} } }}\left[ {\frac{{\sqrt {t_{j} \rho_{j} } - \sqrt {t_{i} \rho_{i} } }}{{t_{j} - t_{i} }}} \right]$$

(2) To find the “quasi” resistivity

16 $$\rho = 4\left[ {\frac{{\sqrt {t_{j} \rho_{j} } - \sqrt {t_{i} \rho_{i} } }}{{t_{j} - t_{i} }}} \right]^{2} t_{ij}$$

(3) To seek depth

17 $$H = 0.441\frac{{\left( {d_{rj} + d_{ri} } \right)}}{2}$$

When the “smoke ring” approximate inversion is performed on the vertical component, $d_{{z\left( {i,j} \right)}}$ denotes the vertical propagation depth of the current loop, shown as follows:

18 $$d_{{z\left( {i,j} \right)}} = \frac{4}{\sqrt \pi }\sqrt {\frac{{\rho_{{\left( {i,j} \right)}} t_{{\left( {i,j} \right)}} }}{{\mu_{0} }}}$$

When the “smoke ring” approximate inversion is performed on the horizontal component, the lateral propagation distance $d_{{{\text{x}}\left( {i,j} \right)}}$ of the current loop should be calculated, namely^29^:

19 $$d_{{{\text{x}}\left( {i,j} \right)}} \approx 0.55\left( {\frac{{SI\rho_{{\left( {i,j} \right)}} }}{\eta }} \right)^{\frac{1}{5}}$$

In Formula (19), $\eta = 0.5\;\;{\text{ nV/m}}^{{2}}$, denoting a typical noise level, where $S$ is the loop area, $\rho_{{\left( {i,j} \right)}}$ is the average earth resistivity.

## Forward simulation

### 3-D forward algorithm verification

**Figure 2:**
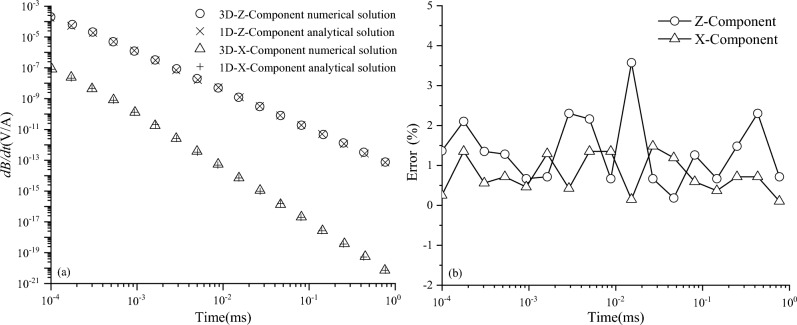
Comparison of calculation results between 3-D FDTD numerical solution and 1-D analytical solution. (**a**) Comparison of induced electromotive force attenuation curves. (**b**) Induced electro-motive force relative error.

By means of a uniform full-space model, the numerical solution obtained by the three-dimensional FDTD forward calculation is compared with the one-dimensional analytical solution in the full space to verify the computational accuracy of the algorithm in this paper^30,31^. The model settings are as follows: the conductivity of the uniform full-space model is 100 Ω m; the transmitter coil is 2 m × 2 m, 10 turns; the overlapping loop device is used; the emission current is 10 A; the current shutdown effect is considered, the turn-off time is 1 μs; and the sampling time is 10^–4^ ms–1 ms. The calculation results are shown in Fig. 2. As can be seen, that in the sampling time range, the response error of the z component and the x component is within 5%, but the response error of the z component is slightly larger than the response error of the x component. Overall, the calculation accuracy of the three-dimensional FDTD algorithm in this paper is in good agreement with the one-dimensional analytical solution, which meets the requirements of simulation calculation.

### Forward uncertainty analysis

In the 3-D TEM forward modeling, uncertainty analysis is crucial, particularly when dealing with the anisotropic electrical conductivity of coal-bearing strata and separation layer water. The anisotropy in electrical conductivity introduces significant challenges in accurately predicting TEM responses.

In coal bearing strata, Coal-bearing strata typically exhibit varying electrical conductivity due to differences in coal composition, seam thickness, and interbedded materials such as shale and sandstone. Similarly, such as separation layer water, can display anisotropic conductivity influenced by factors like pore structure, fluid content, and geological formations, these variations can introduce substantial uncertainty into the model.

To address these uncertainties, one commonly used approach is to conduct lithological physical experiments to determine average conductivity values. This method simplifies the complexity by averaging the conductivity over different directions, thus providing a more manageable, albeit less detailed, representation of the subsurface properties. While this simplification helps in reducing the computational burden and stabilizing the model, it inherently overlooks the finer details of anisotropic variations.

Despite its simplicity, using average conductivity values derived from physical experiments is a practical solution for assigning model parameters. It allows for a more straightforward implementation of 3D TEM forward modeling while acknowledging the inherent uncertainties^32^. To enhance robustness, stochastic modeling techniques, such as Monte Carlo simulations, can be employed. These techniques generate multiple realizations accounting for anisotropic variations and identify key parameters influencing the model. By combining these approaches, the uncertainty in anisotropic conductivity can be effectively managed, leading to more reliable subsurface interpretations.

### Establishment of aquifer model of mine separation layer

After the coal seam is mined, the coal measure stratum is damaged to varying degrees by the mining, which is manifested as uneven and asynchronous deformation and subsidence. Accordingly, a separation space is easily formed between the soft and hard rock layers. The electrical characteristics of the separation space are different from those of the surrounding rock, displaying clear temporal and spatial evolution characteristics. In a short period of time, the abscission space is inflated. At this time, the abscission space exhibits high resistance while the surrounding rock displays relatively low resistance. After a period, under the influence of mining action and roof rock pressure, the overlying rock is deformed and broken, and consequentially a large number of cracks are formed. Currently, the abscission space is characterized by low resistance; in contrast, the surrounding rock is characterized by relatively high resistance. On this basis, the development position, water-richness, and boundary range of the abscission can be determined^11,33^.

Although the aquifers display the general electrical characteristics of water, their geophysical characteristics are different from those of water-conducting faults, gobs, water-filled subsidence columns, and aquifers. The specific manifestations are as follows: (1) When there is a water-conducting fault, the resistivity contour is basically a straight line and relatively dense in the fault area; the resistivity value decreases sharply with the increase of depth. After passing through the fault, the resistivity contour gradually becomes sparse, and the resistivity value continues to increase uniformly with the increase of depth. (2) When there is stagnant water in the goaf, the apparent resistivity contour will bend or even close in the area where the goaf is located, and the resistivity value here is significantly lower than that of other areas. When there is no stagnant water in the goaf, the resistivity value is obviously higher than that of other areas. (3) When the collapsed column is not filled with water or does not conduct water, the structure is relatively complete, and the electrical characteristics are high resistance. When the internal structure of the collapsed column is relatively chaotic and pores and fissures are highly developed but not filled with water, the performance is as follows: High resistance characteristics; if the collapsed column is filled with water, the electrical characteristics are featured with relatively low resistance. (4) When there is an aquifer, the apparent resistivity profile will be banded in the lateral direction, and the apparent resistivity value is obviously lower than that of other areas.

**Figure 3:**
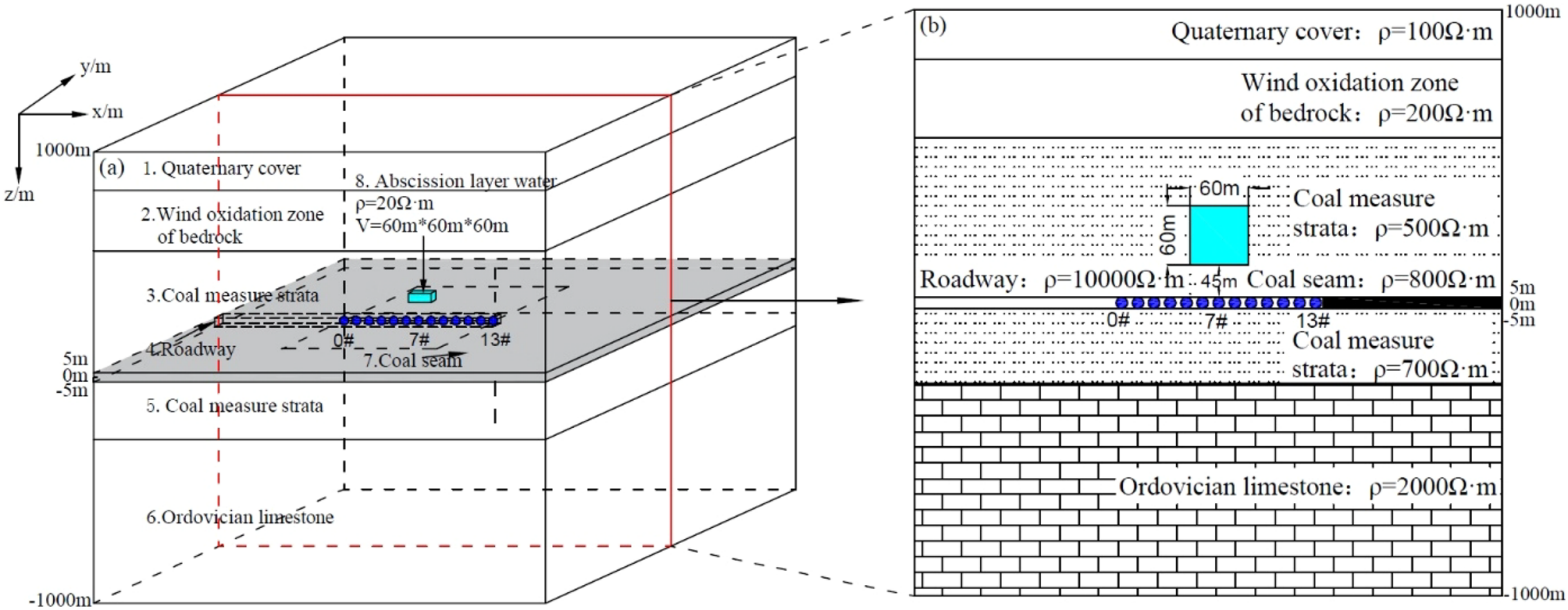
3-D geologic and geophysical model (3-D forward modeling based on resistivity logging data). (**a**) Map of 3-D geological model. (**b**)The main view of the model.

With the regional geological development sequence in a certain study area as the object, a typical geological-geophysical model of separation layer water was established. The geological-geophysical model is simplified into 6 strata, 1 separation layer aquifer and 1 roadway. As shown in Fig. 3, the first layer is the Quaternary overburden with a depth of 1000–800 m; the second layer is the bedrock wind oxidation zone, with a depth of 800–500 m; the third to fifth layers are coal-measure strata. The lithology is mainly sand, mudstone inter-beds and coal seams, with depths of 500–200 m, 200–100 m and 100–300 m, of which 100–0 m is coal seam; the sixth layer is Ordovician limestone layer, with a depth of 300–1000 m. The aquifer size is set to 60 m × 60 m × 60 m, and the geometric center of the aquifer is 75 m away from the roadway roof.

According to the statistics of resistivity logging data^34^, and with comprehensive consideration for the electrical characteristics of formation lithology in the study area, the resistivity of the Quaternary overburden is set at 100 Ω m; that of the bedrock wind oxidation zone is set at 200 Ω m; that of the roof coal-measure stratum is set at 500 Ω m; that of the floor coal-measure stratum is set at 700 Ω m; that of the coal seam is set at 800 Ω m; the Ordovician limestone layer is extremely weak and water-rich, and its resistivity is set at 2000 Ω m; the resistivity of the aquifer is set at 20 Ω m; the roadway for simulation calculation is filled with air and its resistivity is set at 10,000 Ω m. The geo-electric parameter settings of the geological-geophysical model are shown in Table 1.

**Table 1:** Geo-electrical parameters.

No.	Stratum	z/*m*	Electrical resistivity /Ω m	Thickness/*m*
1	Quaternary overburden	1000 to 800	100	200
2	Bedrock wind oxidation zone	800 to 500	200	300
3	Coal-measure stratum	500 to 0	500	500
4	Roadway	5 to − 5	10,000	10
5	Coal-measure stratum	0 to − 300	700	300
6	Ordovician limestone	− 300 to − 1000	2000	700
7	Coal seam	100 to 0	800	100
8	Separation layer aquifer	105 to 45	20	60

### The parameter setting of forward modeling and the establishment of observation system

The 3-D FDTD numerical calculation method is used to simulate the forward modeling of mine transient electromagnetic. The transmitter coil is set at 2 m × 2 m, 10 turns, and the emission current is 10 A. The influence of the current shutdown effect on the secondary field is ignored, and the sampling time is 10^–4^ ms–1 ms logarithmically equally divided into 60 sampling time points. The overlapping loop device is used to detect on the roadway roof (as shown in Fig. 3). Along the roadway roof, a survey line is arranged from (0, 0, 0) to (240, 0, 0) with a total length of 240 m. A total of 13 measuring points are set at an interval of 20 m.

### Analysis of the response characteristics of water body

**Figure 4:**
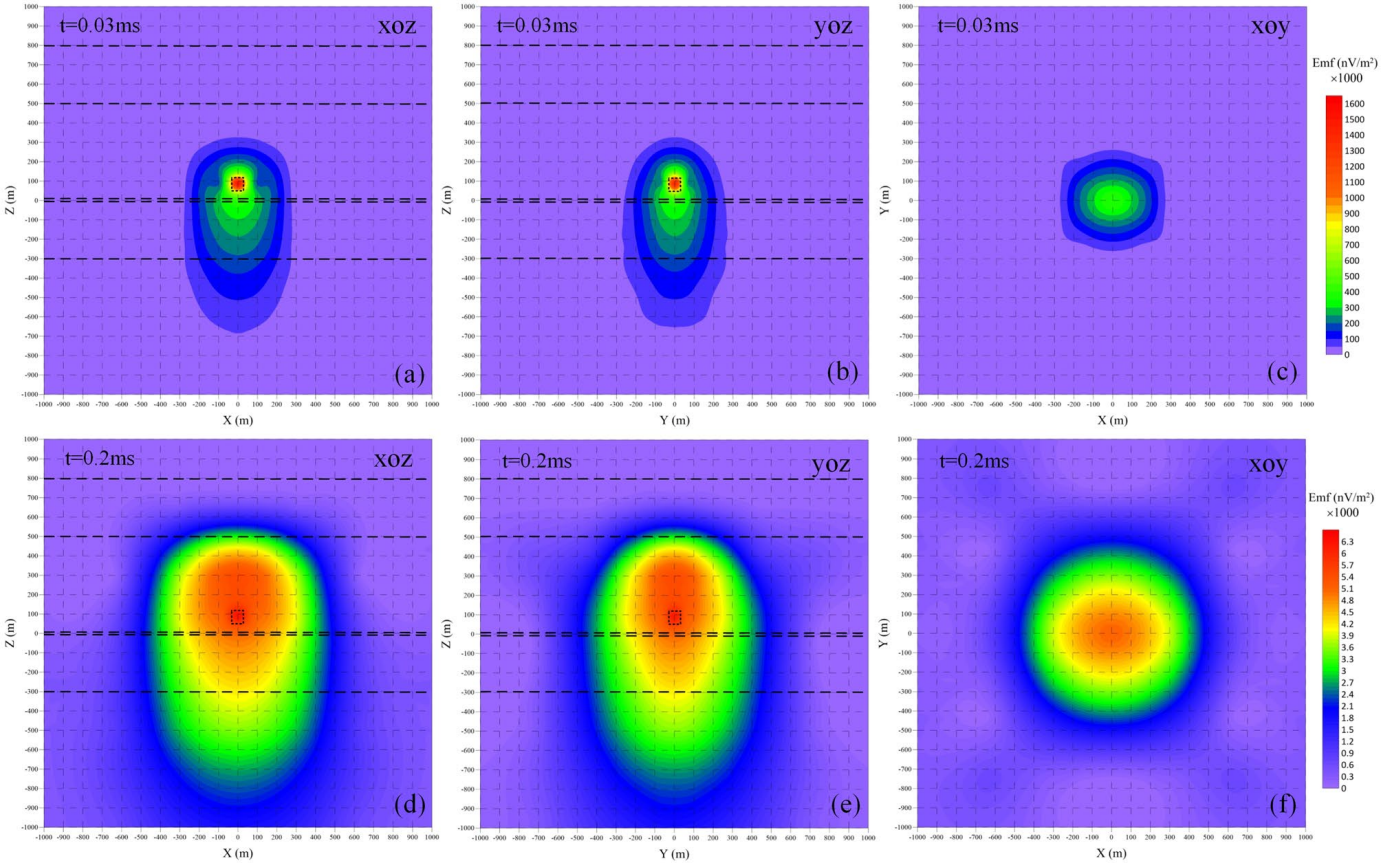
Emf contour map for 3D forward modeling. (**a**) xoz slice with t = 0.03 ms. (**b**) yoz slice with t = 0.03 ms. (**c**) xoy slice with t = 0.03 ms. (**d**) xoz slice with t = 0.2 ms. (**e**) yoz slice with t = 0.2 ms. (**f**) xoy slice with t = 0.2 ms.

The 3-D FDTD forward calculation was performed on the roof water body model in Fig. 3, and the results of the induced electromotive force (Emf) contour are shown in Figs. 4. The calculated Emf is along the three directions of xoz, yoz, and xoy. Slicing was performed, and two-dimensional contour maps of Emf were obtained at 0.03 ms and 0.2 ms, respectively. As shown in Fig. 4a or d, at the moment of 0.03 ms, the primary eddy current field excited by the coil spreads outward with the center of the coil as the origin, and the primary eddy current field induces a new eddy current field at the position of the low-resistance body. The new eddy current field intensity is concentrated in the low-resistance body, and the diffusion shape is approximately a semi-ellipse. With the delay of sampling time, the new eddy current field intensity spreads and decays around the model with the low resistance body as the center. At 0.2 ms, the eddy current field intensity takes the coal seam/roadway as the boundary, and the intensity value distribution range of the roof is obviously larger than that of the bottom plate; and the overall diffusion range is semi-elliptical. It is inferred that the resistivity of the Quaternary overburden and the bedrock wind oxidation zone is generally low, thus forming a relatively low-resistance shielding layer in the shallow part. Figure 4b or e is the slice diagram of the Emf contour in the yoz direction. Similar to the xoz direction slice, the yoz direction slice is perpendicular to the plane where the emission source is located. It can be seen from Fig. 4b or e that the Emf distribution law in the yoz direction is the same as that in the xoz direction. The distribution laws are basically the same, thus, not to be repeated for the sake of simplicity. Figure 4c or f is a slice diagram of the Emf contour in the xoy direction. As can be seen, the slice in the xoy direction is parallel to the plane where the emission source is located. The shape is similar to a circle. With the delay of time, at 0.2 ms, the primary eddy current field spreads around the model with the center of the coil as the origin, and the diffusion shape is similar to a circle. A new eddy current field is formed at the position of the body, which indicates that the diffusion velocity of the primary eddy current field in the plane parallel to the emission source is lower than that of the plane perpendicular to the emission source.

In order to further show the three-dimensional transient electromagnetic single-component response characteristics of the low-resistance body, the curves of the induced electromotive force of the vertical and horizontal components at each measuring point which decay with time were drawn respectively. On this basis, the corresponding apparent resistivity curves were drawn accordingly.

**Figure 5:**
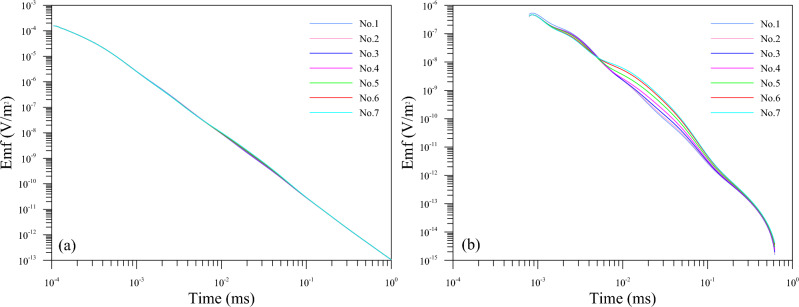
Emf curves at different measuring points (**a**) vertical component, (**b**) horizontal component.

Figure 5a is the induced electromotive force curve of the vertical component at each measuring point; Fig. 5b shows the induced electromotive force curve of the horizontal component at each measuring point. As can be seen, the overall response of the induced electromotive force decay curve of the vertical component is not obvious. The induced electromotive force curves at the early measuring points tend to be consistent; as for the secondary eddy current field of the low-resistance body, weak response can be observed at 0.01 ms–0.1 ms. After the secondary eddy current field formed by the low-resistance body is gradually diffused, the induced electromotive force curves at each measuring point tend to be consistent. The induced electromotive force curve of the horizontal component has a stronger response to the secondary eddy current field generated by the low resistance body at 0.01 ms–0.1 ms than the case of the vertical component. The induced electromotive force decay curve received during this period declines slowly, with a large degree of separation and more obvious low resistance response. The specific performance is that the closer the measurement point is to the geometric center of the low-resistance body, the slower the attenuation and the greater the separation, the more obvious the response.

**Figure 6:**
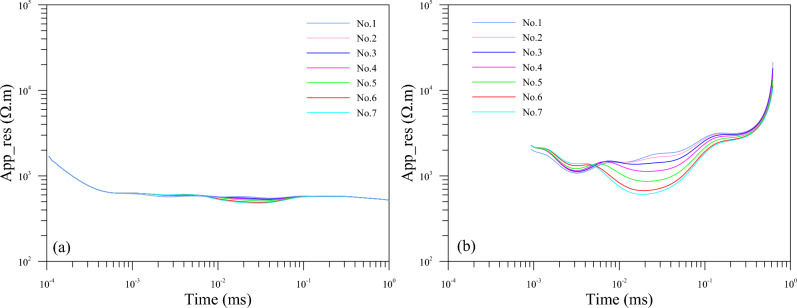
The late apparent resistivity curves at different measuring points. (**a**) Vertical component. (**b**) Horizontal component.

Figure 6a displays the apparent resistivity curve of the vertical component at each measuring point. Figure 6b is that of the horizontal component at each measuring point. As can be seen, the reflection of the low-resistance body can be observed at 0.01 ms–0.1 ms in the apparent resistivity curve of the vertical component obtained the late period. The late apparent resistivity curves of the early tends to be consistent with that of late measuring points. The apparent resistivity curve of the horizontal component in the late period has a stronger reflection on the low-resistance body than that of the vertical component at 0.01 ms–0.1 ms. The specific performance is that the closer the measuring point is to the geometric center of the low-resistance body, the more obvious the low-resistance reflection.

**Figure 7:**
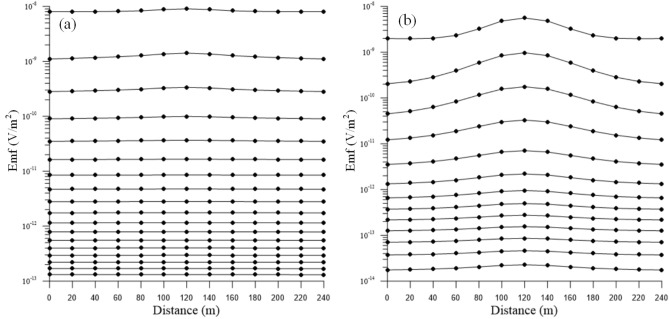
Multi-track curves for induced voltage (**a**) vertical component. (**b**) Horizontal component.

Figure 7 shows the multi-track response curves of 13 measuring points obtained by 3-D FDTD forward modeling, in which Fig. 7a is the multi-track curve of vertical component, and Fig. 7b is that of horizontal component. As can be seen from Fig. 7, the multi-track response curve of the vertical component is relatively flat as a whole. The multi-track curve at the No. 7 measuring point (corresponding to 120 m on the abscissa) in the early sampling period shows a low-resistance body response that is slightly lifted upward, but the response amplitude is small and insignificant. The multi-track curve in the late sampling period is almost a straight line, and the response to the low-resistance overlay in the model is weak. The multi-track response curve of the horizontal component rises upward as a whole, and the peak response amplitude appears at the No. 7 measuring point, which corresponds to the position of the geometric center of the low-resistance body in the 3-D model. The multi-track curve in the early sampling period shows a significant upward lift, indicating that the low-resistance body response amplitude is large and obvious. The multi-track curve in the late sampling period tends to lift upwards slightly, and the response to the low-resistance overlay in the model is weak.

**Figure 8:**
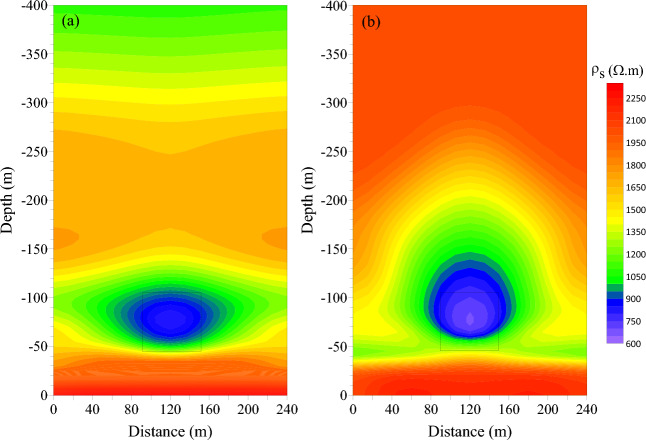
Comparison of late apparent resistivity profiles of vertical and horizontal components. (**a**) Vertical component. (**b**) Horizontal component.

**Figure 9:**
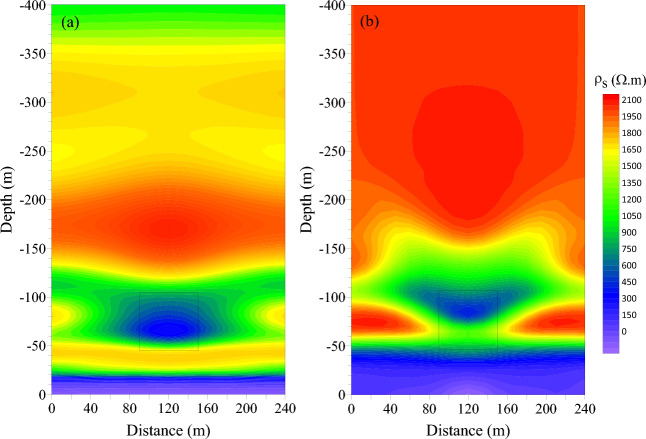
Comparison of “smoke ring” inversion profiles in vertical and horizontal components. (**a**) Vertical component. (**b**) Horizontal component.

In order to further highlight the three-dimensional transient electromagnetic response characteristics of the low-resistance body in the roadway roof, the late apparent resistivity solution and one-dimensional “smoke ring” approximate inversion process was conducted with the three-dimensional forward data of the vertical and horizontal components. Figures 8 and 9 show the late apparent resistivity profile of the component and the horizontal component and the approximate inversion profile of the “smoke ring”.

Among them, Fig. 8a displays the late apparent resistivity profile of the vertical component, and Fig. 8b is that of the horizontal component. As can be seen, the late apparent resistivity profile of the vertical component is well layered along the vertical direction. The layered characteristics along the lateral direction are obvious, which is in line with the characteristics of the coal measure strata, indicating that the vertical component has a high vertical resolution of the strata. The response to the low-resistance body is obvious, and the shape is similar to an ellipse, which is obviously different from that of the actual model. The late apparent resistivity profile of the horizontal component is poorly layered along the vertical direction, featured with an obvious response to the low-resistance body. The resolution is good, and this advantage can be fully utilized in the actual data processing process to make up for the shortcoming of low resolution response to low-resistance bodies.

Figure 9a shows the inversion profile of the “smoke ring” in the vertical component, and Fig. 9b is that of the “smoke ring” in the horizontal component. As can be seen, the “smoke ring” inversion profile in vertical component is well layered along the vertical direction. The layered characteristics in the lateral direction are obvious, which are in good agreement with the characteristics of coal measure strata, indicating a higher vertical resolution of the vertical component on the stratum. The inversion of the low-resistance body is more obvious, but the shape of the low-resistance body is different from the actual model. The “smoke ring” inversion profile in horizontal component is poorly layered along the vertical direction, and the inversion of the low-resistance body has a more obvious response; but the shape of the low-resistance body is quite different from that of the actual model. This indicates that the “smoke ring” approximate inversion method displays a better response to the low-resistance body, but poor resolution of the boundary of the low-resistance body.

## Engineering example

### The measurement of roof water body and the establishment of forward modeling model

**Figure 10:**
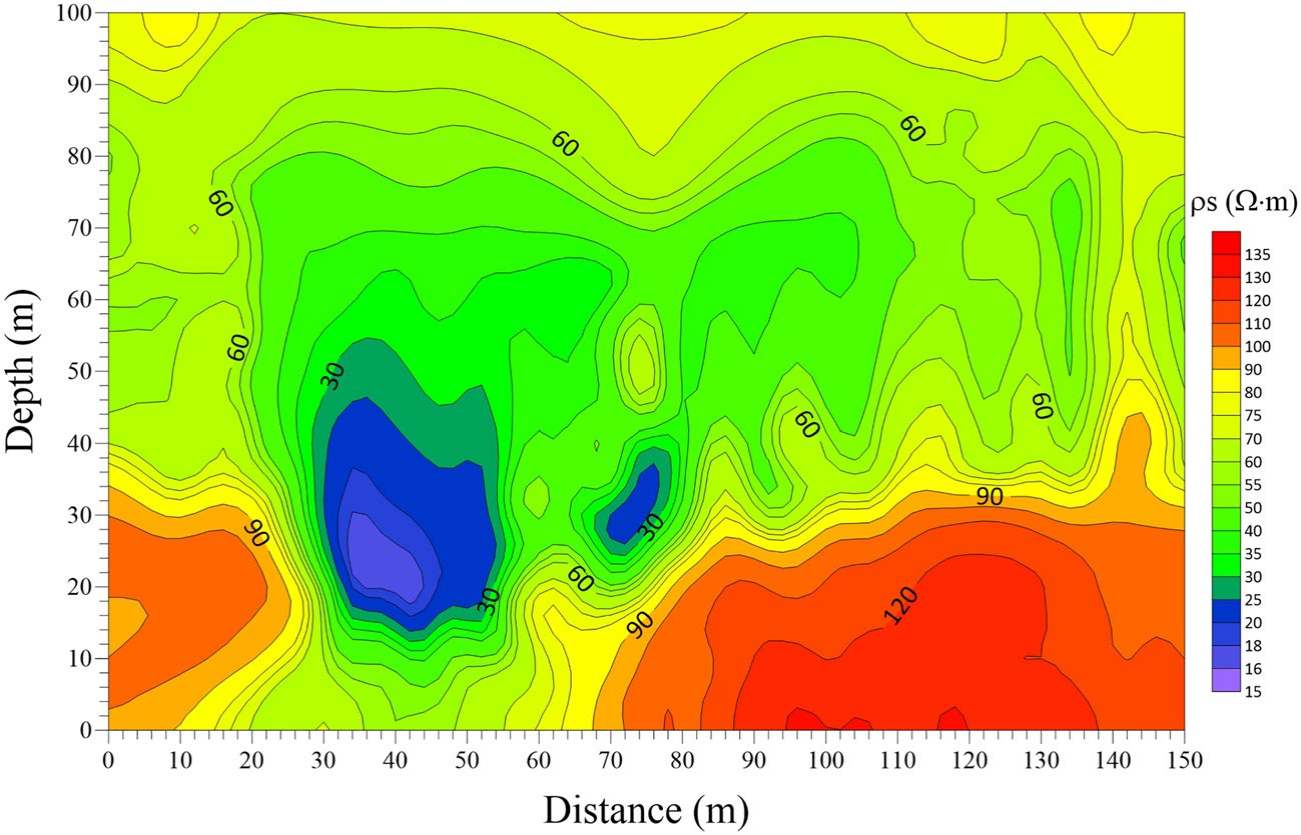
The pseudo-profile view of apparent resistivity of the measured transient electromagnetic data.

**Figure 11:**
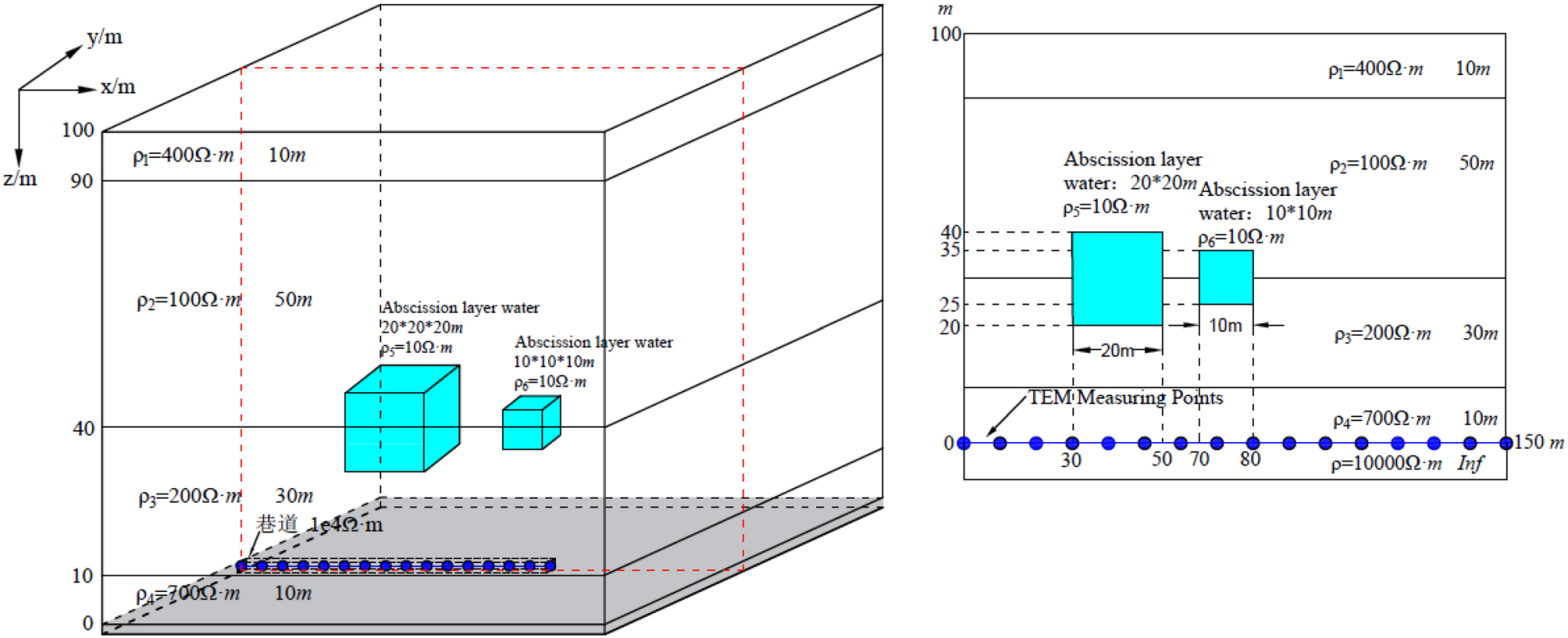
Main view of 3-D forward model (A 3-D forward modeling geological-geophysical model can be established according to the electrical structural characteristics in Fig. 10).

In a certain mine, during the mining process of the lower group coal, overlying water gushing occurred. According to the analysis of hydrogeological data, the source is very likely to be separation layer water. Since then, the mine has strengthened the detection of the roof overlying rock. Figure 10 shows a pseudo-profile view of the apparent resistivity of the measured transient electromagnetic data of the roof in this mine. As is shown in Fig. 10, the apparent resistivity profile is well layered in the vertical direction, and the apparent resistivity value exhibits a high-low–high variation in the depth direction. There are two obvious relatively low-resistance anomaly areas: one is at a horizontal distance of 30–50 m and a depth of 15–40 m; the other is at a horizontal distance of 70–80 m, and a depth of 25–35 m. This is presumed to be caused by the separation layer water in the roadway roof. Since the electrical characteristics of the roof water-rich anomaly are similar, a 3-D forward modeling geological-geophysical model can be established according to the electrical structural characteristics in Fig. 10. The parameter settings of the model are shown in Fig. 11. In total, the 3-D forward modeling model has four layers. In addition, a high-resistance layer is set under the top plate, with an infinite thickness, simulating a high-resistance air layer, which does not affect the actual detection. The resistivity parameter setting follows a high-low–high variation with depth. Two low-resistance anomalies are respectively set at two points: one is at a horizontal distance of 30–50 m and a depth of 20–40 m, and the other at a horizontal distance of 70–80 m and a depth of 25–35 m. The resistivity of the low-resistance anomaly is set at 10 Ω m. A measuring line is arranged along the roadway roof, with a total length of 150 m. A total of 16 measuring points are set at an interval of 10 m.

### 1-D forward modeling response features

**Figure 12:**
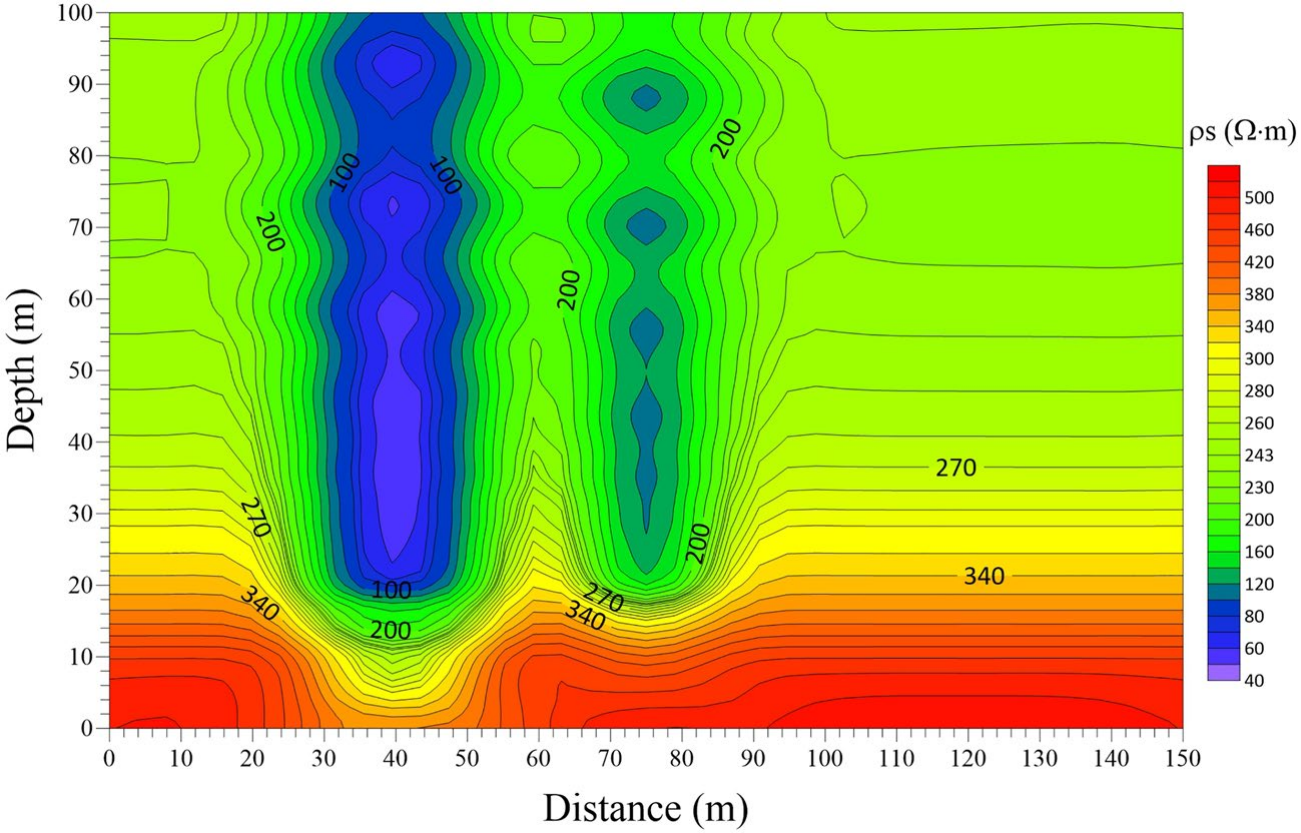
Apparent resistivity profile obtained by 1-D full-space forward modeling method.

The model in Fig. 11 is calculated by the one-dimensional full-space forward modeling method, and Fig. 12 displays the apparent resistivity profile calculated by the one-dimensional full-space forward modeling. As the depth increases, the apparent resistivity value presents a high-low variation characteristic. There are two obvious strip-shaped relatively low-resistance anomalies: one is at a horizontal distance of 30–50 m and a depth of 20–100 m; the other is at a horizontal distance of 70–80 m and a depth of 25–100 m. Although the forward modeling response has good lateral resolution of the two low-resistivity anomalies in the model, there is a significant deviation in the vertical resolution of the anomaly. This is because the one-dimensional forward method only records the induced electromagnetic field information from the vertical direction when the complex abnormal body model with low resistance is observed. When one of the two adjacent measuring points in the vertical direction passes through the low-resistance body while the other does not, the former displays a low-resistance response in the vertical direction, and the latter can be taken as the model background field response. Accordingly, when the data of the two measuring points are interpolated, a strip-shaped low-resistance anomaly would be formed, as shown in Fig. 12.

### 3-D forward response features

**Figure 13:**
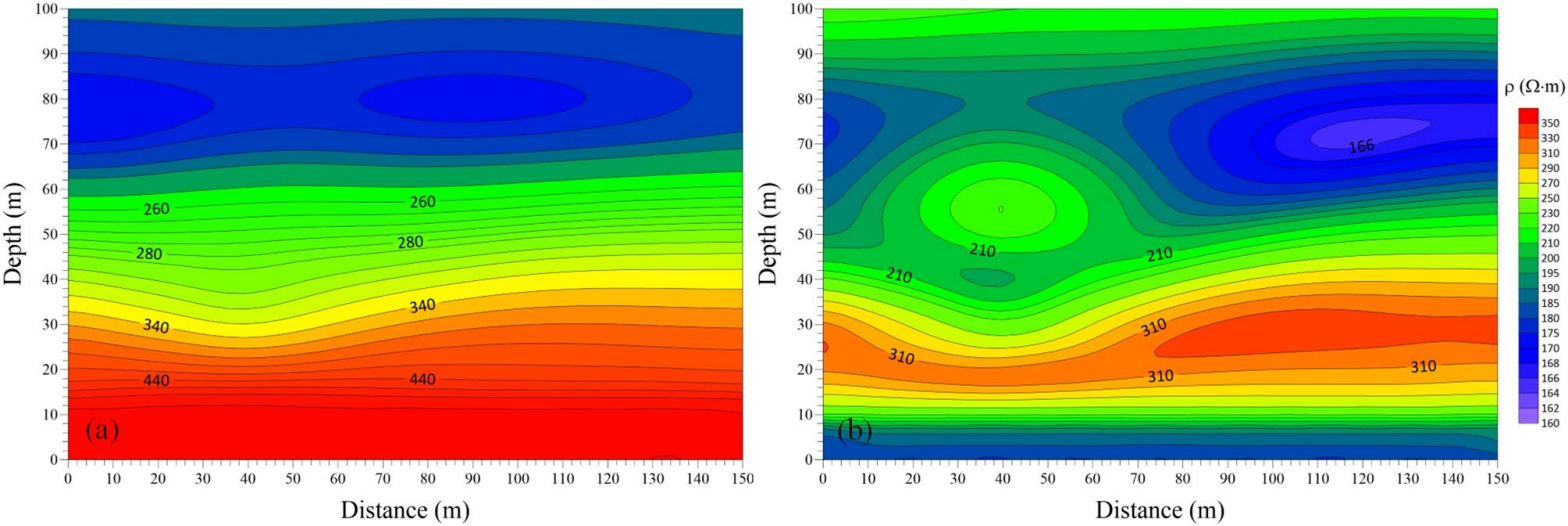
Profiles of apparent resistivity and “smoke ring” approximate inversion of vertical component in the case of 3-D forward modeling. (**a**) Apparent resistivity profile. (**b**) “Smoke ring” inversion profile.

**Figure 14:**
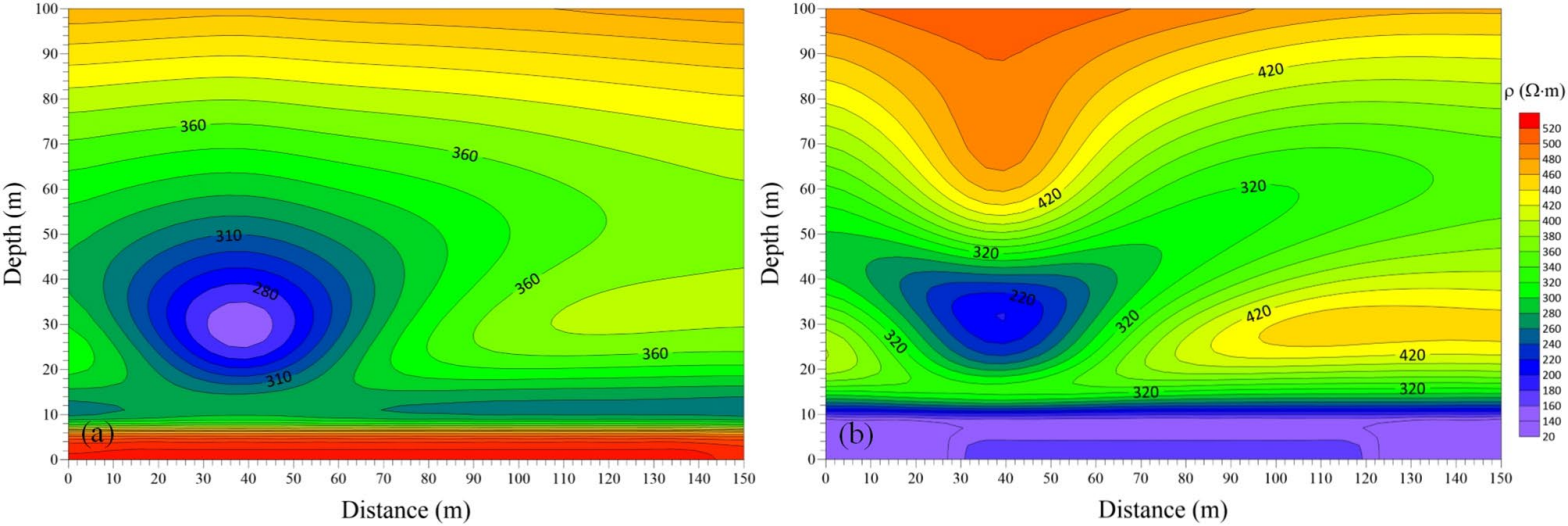
Profiles of apparent resistivity and “smoke ring” approximate inversion of horizontal component in the case of 3-D forward modeling. (**a**) Apparent resistivity profile. (**b**) “Smoke ring” inversion profile.

The model in Fig. 11 was calculated by using the 3-D FDTD forward modeling method. The late apparent resistivity solution and the one-dimensional “smoke ring” approximate inversion were conducted on the 3-D forward modeling data of the vertical and horizontal components. The profiles of late apparent resistivity and “smoke ring” approximate inversion of the vertical and horizontal components are drawn and shown in Figs. 13 and 14 respectively. Specifically, Fig. 13a shows the late apparent resistivity profile of the vertical component, and Fig. 13b is the “smoke ring” approximate inversion profile of the vertical component. As is shown in Fig. 13a, the apparent resistivity displays a good stratification. With the increase of the detection depth, the apparent resistivity value shows a high-low variation, with no obvious response to the low-resistance body. The apparent resistivity electrical profile of vertical component differs greatly from the 3-D forward model. As is shown in Fig. 13b, the resistivity of the “smoke ring” approximate inversion is relatively continuous in the lateral direction. With the increase of the detection depth of the roof, the vertical resistivity value shows a low–high–low–high variation. The electrical interface presents a concave feature at a horizontal distance of 20–50 m and a depth of 30–40 m, which denotes a weak response of a low-resistance body. On the whole, the “smoke ring” inversion electrical profile of the vertical component differs greatly from the three-dimensional forward modeling model, displaying a poor inversion effect.

Figure 14a shows the late apparent resistivity profile of the horizontal component, and Fig. 14b is that of the “smoke ring” approximate inversion. As is shown, the apparent resistivity is well layered in the vertical direction. With the increase of the detection depth of the roof, the vertical apparent resistivity value shows the characteristics of high–low–high variation. An obvious relatively low-resistance anomaly area appears at a horizontal distance of 20–60 m and a depth of 20–50 m, with no obvious response to another low-resistance anomaly. This indicates that the apparent resistivity electrical profile of the horizontal component can well reflect the electrical structure of 3-D forward modeling model. As is shown in Fig. 14b, the resistivity of the “smoke ring” approximate inversion is relatively continuous in the lateral direction. With the increase of the detection depth of the roof, the vertical resistivity value displays a low–high–low–high variation. An obvious relatively low-resistance anomaly area appears at a horizontal distance of 20–60 m and a depth of 20–50 m, with no obvious response to another low-resistance anomaly. On the whole, the electrical profile of the “smoke ring” approximate inversion of the horizontal component is basically consistent with the 3-D forward modeling model.

## Conclusion

This paper aims at the geological disasters caused by the aquifer in the mine roof. This paper used the overlapping small loop device to simulate the 3-D time-domain finite difference forward modeling. The diffusion law of the transient electromagnetic field in the aquifer of the roof was analyzed and summarized. On this basis, the following conclusions were drawn:

The existence of separation layer water has a significant impact on the diffusion speed of induced electromotive force. To be specific, the diffusion speed of induced electromotive force parallel to the plane where the emission source is located is smaller than that of the plane perpendicular to the emission source. Compared with the case in the vertical direction, the decay curve of induced electromotive force in the horizontal direction displays a much greater response intensity to the separation layer water. The closer the measuring point is to the geometric center of the low-resistance body, the slower the attenuation and the greater the separation will be similarly, the more obvious the response is.

The horizontal component of apparent resistivity displays a high response resolution to the electrical reflection of aquifers and the identification of separation layer water. This finding provides a theoretical basis for the development of mine transient electromagnetic three-component detection instruments and the process of three-component data processing, inversion, and interpretation. This advantage can be fully utilized to offset the shortcomings of conventional single-component transient electromagnetic detection instruments with low response resolution to separation layer water.

With the three-dimensional model established by measuring the apparent resistivity electrical profile of the separation layer water, one-dimensional full-space forward modeling and three-dimensional forward modeling were conducted. As is revealed, the one-dimensional full-space forward modeling displays better lateral resolution for the complex separation layer water model but is greatly affected by the observation method and data interpolation. In contrast, the vertical component of the 3-D forward model displays a weaker response to the aquifer than the horizontal component. Accordingly, the apparent resistivity electrical profile of the horizontal component can well reflect the electrical properties of the 3-D forward model, which is consistent with the results of numerical simulation.

## Data Availability

The datasets used and/or analyzed during the current study available from the corresponding author on reasonable request.
